# Long intergenic non-protein coding RNA 1273 confers sorafenib resistance in hepatocellular carcinoma via regulation of methyltransferase 3

**DOI:** 10.1080/21655979.2022.2025701

**Published:** 2022-01-17

**Authors:** Huifang Kong, Jie Sun, Wei Zhang, Huixin Zhang, Hong Li

**Affiliations:** a The First Ward of Hepatology Department, Fifth Medical Center of Chinese Pla General Hospital, Beijing, China; b Internal Medicine- Fifth Medical Center of Chinese PLA General Hospital, Beijing, China; c Department of Infectious Diseases, The Affiliated Hospital of Guizhou Medical University, Guiyang, Guizhou Province, China

**Keywords:** Hepatocellular carcinoma, LINC01273, N6-methyladenosine, sorafenib resistance

## Abstract

Hepatocellular carcinoma (HCC) is often diagnosed in patients with advanced disease who are ineligible for curative surgical therapies. Sorafenib is a first-line agent approved for the treatment of advanced HCC. However, the frequent resistance of HCC cells to sorafenib greatly reduces its efficacy. Herein, we describe a novel long non-coding RNA (lncRNA) conferring sorafenib resistance. Long intergenic non-protein coding RNA 1273 (LINC01273) was significantly overexpressed in human HCC and sorafenib-resistant tissues, linking it to poor overall and relapse-free survival. We established sorafenib-resistant Huh7 (Huh7-SR) and SMMC-7721 (SMMC-7721-SR) cells, and found that the knockdown of LINC01273 repressed the viability, colony formation, and DNA synthesis rate of Huh7-SR and SMMC-7721-SR cells. The level of N6-methyladenosine (m^6^A) in sorafenib-resistant HCC cells was significantly decreased, which was rescued by LINC01273 silencing. Mechanistically, LINC01273 complementarity bound to miR-600, served as a ‘reservoir’ increasing miR-600 stability, and facilitating miR-600 targeting methyltransferase 3 (METTL3), a m^6^A ‘writer’, resulting in reducing METTL3 level. In addition, LINC01273 was modified with m^6^A, METTL3 increased LINC01273 m^6^A modification, followed by LINC01273 decay in the presence of YTHDF2, a m^6^A ‘reader’. Our findings reveal the key role of LINC01273 in sorafenib-resistant HCC cells, and targeting of the newly identified LINC01273/miR-600/METTL3 feedback regulatory axis may be a promising effective intervention for HCC patients with sorafenib resistance.

## Introduction

Primary liver cancer is the sixth most common cancer in the world, and the third-leading cause of cancer death in 2020, with about 906,000 new cases and 830,000 deaths [1]. Hepatocellular carcinoma (HCC) is the main subtype of primary liver cancer, accounting for 75-85% [2]. Compared with its incidence, HCC has a relatively higher mortality rate, due to 80% of the patients who had entered the advanced stage when they were first diagnosed, the chance of radical surgery has been lost [3]. Even with radical surgical treatment, 60-70% of patients still develop metastasis and recurrence within 5 years [4]. The median survival time of patients with advanced HCC is only about 1 year, and the 5-year survival rate is just as low as 10.1% [5]. Sorafenib is the first first-line drug approved for the treatment of advanced HCC, but many HCC patients respond poorly to sorafenib or develop resistance months after treatment [6]. Therefore, there is an urgent need to elucidate the mechanism of sorafenib resistance in order to use this drug more effectively for HCC patients.

Long non-coding RNA (lncRNA) is a class of eukaryotic non-coding RNA molecules whose length is greater than 200 nt [7]. It was first discovered as ‘dark matter’ in the product of gene expression. Recent studies have shown that lncRNAs play a role in biological processes such as disease occurrence, cell cycle and stem cell differentiation, and the related molecular mechanisms can be roughly divided into four types: signal, bait, guide, and scaffold molecules [8]. Due to the large number of lncRNAs and the complex mechanism of action, the functions of many lncRNAs are still unknown and need to be further studied. Emerging evidence suggests that lncRNA is closely related to drug resistance of human cancers [9], for example, H19 was upregulated in breast cancer and high H19 conferred cancer cell resistant to doxorubicin [10]. TINCR was significantly increased in trastuzumab-resistant breast cancer cells and enhanced trastuzumab resistance via inducing epithelial-mesenchymal transition [11]. Exosomal-derived APCDD1L-AS1 induces 5-fluorouracil resistance in oral squamous cell carcinoma via acting as a miRNA sponge [12]. Whether lncRNA is involved in sorafenib resistance remains largely unknown.

N6-methyladenosine (m^6^A) is the most common type of RNA methylation in eukaryotes, accounting for more than 80% [13]. There are three types of modifiers for m^6^A: m^6^A methyltransferase, also known as m6A ‘writer’, includes METTL3, METTL14, WTAP, and other related proteins, whose main function is to catalyze RNA methylation [14]; m^6^ A demethylase (‘writer’), such as ALKBH5 and FTO binding proteins can reverse the methylation modification that has been formed [15] m^6^ A ‘reader’ is a methylated recognition protein, mainly involved in the YTH domain of RNA-binding protein (YTHDF1, YTHDF2, YTHDF3, YTHDC1, and YTHDC2), with main role binding selective m^6^A sites, thus affecting RNA splicing, localization, translation, stability, and so on [16]. These proteins involved in the m^6^A process are often deregulated in human cancer and are closely associated with cell malignant behaviors [17,18]. Among them, METTL3, as the best known m^6^A methyltransferase, has been identified as a critical regulator in multiple biological processes, including cell cycle, apoptosis, migration, invasion, differentiation, and inflammatory response [19]. Recently, METTL3 was reported as a negative factor of sorafenib resistance in HCC, it restored sorafenib sensitivity via autophagy in a m^6^A-dependent manner [20]. However, how METTL3 is regulated in sorafenib-resistant HCC cells is still unknown.

Herein, by analyzing Gene Expression Omnibus (GEO) database, we found a novel lncRNA involved in sorafenib resistance of HCC cells. LINC01273 was significantly upregulated in HCC and sorafenib-resistant tissues. Further study found that LINC01273 reduced sorafenib sensitivity of HCC cells by regulation of miR-600/METTL3 axis. Besides, LINC01273 was also controlled by m^6^A modification mediated by METTL3. The deregulation of LINC01273/METTL3 feedback axis may be critical for the sorafenib resistance in HCC. Therefore, in the present study, we aim to investigate the role of LINC01273 in sorafenib-resistant HCC, and further reveal its potential regulatory mechanism, in order to provide new therapeutic targets and strategies for HCC patients with sorafenib resistance.

## Materials and methods

### HCC samples

A total of 105 pairs of HCC and adjacent normal tissues were collected from The Affiliated Hospital of Guizhou Medical University. The detailed features of HCC patients are shown in Table 1. Patients who had received anti-tumor treatment other than sorafenib before surgery were excluded. Sorafenib resistance existed in 29 out of 105 HCC patients. Sorafenib resistance criteria are as follows: HCC patients were evaluated as progressive patients according to the modified solid tumor efficacy evaluation standard (mRECIST) after oral sorafenib [21]. Patients were routinely followed up after discharge, and all patients signed informed consent after being informed of the specific experimental protocol, which was reviewed and approved by the Medical Ethics Committee of the Affiliated Hospital of Guizhou Medical University (ZNO-1750396).

**Table 1.** The clinicopathologic features of HCC patients (n = 105)

Features	Case
Age	
≤ 50	46 (43.8%)
> 50	59 (56.2%)
Gender	
Male	82 (78.1%)
Female	23 (21.9%)
AFP (ng/ml)	
≤ 400	17 (16.2%)
> 400	88 (83.8%)
Tumor diameter (cm)	
≤ 5	41 (39.1%)
> 5	64 (60.9%)
Vascular invasion	
No	22 (20.9%)
Yes	83 (79.1%)
Edmondson	
III–IV	105 (100%)
Sorafenib	
Resistance	29 (27.6%)
Sensitive	76 (72.4%)

### qRT-PCR assay

Total RNA was extracted from Trizol (Cat.15596026, Invitrogen, Carlsbad, CA, USA), and cDNA was synthesized by using SuperScript™ III reverse transcriptase (Cat.18080093, Invitrogen). Real-time PCR (qPCR) was performed with SYBR Green real-time PCR master mixes (Cat.K0221, Invitrogen) according to the manufacturer’s instructions. Gene relative expression was calculated using 2^–ΔΔCT^ method, and GAPDH was used as reference control. The primer sequences are as follows:

LINC01273: Forward: 5`-TTAGAGGTGGCAGTGGCTCT-3`, Reverse: 5`-CCCTCGCGTTAATCACTGTT-3`;

METTL3: Forward: 5`-GCCACTCAAGATGGGGTAGA-3`, Reverse: 5`-GAGAGCTTGGAATGGTCAGC-3`;

GAPDH: Forward: 5`-ACCCAGAAGACTGTGGATGG-3`, Reverse: 5`-TTCAGCTCAGGGATGACCTT-3`;

U6: Forward: 5`-GTTGGAGAGGACCATGGAGA-3`, Reverse: 5`-CACACCAAGGGCAGAAAACT-3`.

### Cell culture and transfection

Huh7 and SMMC-7721 cells were purchased from the American Type Culture Collection (ATCC) and cultured in the Gibco RPMI 1640 medium supplemented with 10% fetal bovine serum and 100 U/ml penicillin-streptomycin solution. Cells were cryopreserved in liquid nitrogen, after resuscitation, and the third passage was used for experiments. Cell transfection was conducted using Lipofectamine 2000 (Cat.11668019, Invitrogen), followed by testing the transfection efficiency using qRT-PCR.

### Generation of sorafenib-resistant HCC cells

HCC cells with sorafenib resistance were established by long-term exposure to sorafenib (Cat.284461–73-0, Selleckchem, Houston, USA). In detail, HCC cells were treated with low doses of sorafenib (0.625 μM) for 2 weeks, followed by cultivation in a fresh, complete medium for another 2 weeks. Then, the dose of sorafenib was gradually increased and the culture mode was followed successively until the dose of sorafenib was increased to 10 μM, the maximum clinically tolerated dose, and the surviving cells were sorafenib-resistant HCC cells.

### Stable knockdown of LINC01273

Four shRNAs targeting LINC01273 (shRNA#1: CCAGAAGAGAAGGGAATAA; shRNA#2:CTGGATGAAAGCTGGAATA; shRNA#3: CATTCCAACACAGACCACA; shRNA#4: CAGCATAAATGCCCAGGAA) were synthesized and inserted into the pLV2-U6-Puro lentiviral vector. Then, psPAX2 packaging plasmid, pMD2G envelope plasmid along with the lentiviral vector was transfected into HEK-293 cells using Lipofectamine 2000. After 48 h, the virus particles were collected and infected into sorafenib-resistant Huh7 and SMMC-7721 cells. The infection efficiency was assessed using qRT-PCR.

### Western blot

The Western blot assay was conducted as previously described [22]. Cells and tissues were lysed in RIPA buffer (Cat.9806, Cell Signaling Technology, Danvers, MA, USA) containing phosphatase inhibitors (Cat.P2850, Sigma, St. Louis, MO, USA) and a protease inhibitor cocktail (Cat.P8340, Roche, Basel, CH). The lysate was subjected to SDS-PAGE, transferred to polyvinylidene fluoride (PVDF) membranes and incubated with primary antibody, followed by HRP conjugated secondary antibody. The band was visualized using SuperSignal™ West Pico PLUS (Cat.34580, Invitrogen). The primary antibodies used are anti-ABCG2 (1:1000 dilution, ab207732, Abcam), anti-METTL3 (1:2000 dilution, ab195352, Abcam), and anti-GAPDH (1:15000 dilution, ab181602, Abcam).

### CCK-8 assay

The cell viability was tested by CCK-8 assay. 100 μL cell suspension containing 2000 cells was added into 96-well plate, and cultured for 24 h, 48 h, and 72 h. During the period, 10 μL CCK-8 reagent (Cat.CK04, Dojindo, Kumamoto, Japan) was added into each well and incubated for 2 h. The light absorption value at 450 nm recorded by a microplate reader indicates cell viability.

### Colony forming assay

Five hundred cells were plated onto 6-well plate and cultured for 2 weeks. The cells were then stained with crystal violet for 10 min, and washed twice by PBS. The number of clones per well was recorded.

### EdU assay

DNA synthesis rate was assessed by using Cell-Light EdU Apollo643 In Vitro Kit as per the supplier’s instructions (Cat.C10310-2, RiboBio, Guangzhou, China). In brief, 1000 cells were plated onto 96-well plate and grew to 70-80% confluency, and EdU reagent was added and incubated for 2 h. After strict washing, the EdU fluorescence signal was observed under microscope.

### Total m^6^A detection and methylated RNA immunoprecipitation (meRIP)

Total m^6^A of HCC cells were tested by m^6^A RNA Methylation Quantification Kit (Colorimetric) (ab185912, Abcam) according to the supplier’s instructions. In brief, total RNA was extracted, and 200 ng RNA was incubated with 80 μL binding solution at 37°C for 90 min, followed by incubation with diluted capture and detection antibodies, respectively. After strict washing, the developer solution was added, and the absorbance was scored on a microplate reader at 450 nm within 15 min. Besides, the meRIP assay was carried out using Magna MeRIP m^6^A Kit (Cat.A-17-10499, Millipore, Schwalbach, Germany) according to the supplier’s protocols. In short, 5 µg total RNA was fragmented for 5 min at 70°C, followed by incubation with 3 µg m^6^A antibody or IgG antibody at 4°C for 6 h. After strict washing, the enriched RNA was eluted and analyzed by qRT-PCR assay.

### RNA pull-down and RIP assays

The RNA pull-down assay was conducted as previously described [23]. In brief, the biotin-labeled anti-sense DNA probes for LINC01273 were designed and synthesized by Sangon (Shanghai, China). The HCC cell lysates were then collected and incubated with LINC01273 probe at for 2 h at 4°C. RNA complexes were washed with NT2 buffer (50 mM Tris-HCl, 150 mM NaCl, 1 mM MgCl_2_, and 0.05% NP-40) and incubated with streptavidin-magnetic C1 beads (Cat.65001, Invitrogen) for 0.5 h at 4°C. The enriched RNA was extracted and used for qRT-PCR analysis. And RIP assay was performed using EZ Magna RNA Immunoprecipitation Kit (Cat.17–701, Millipore) according to the manufacturer’s guidelines. The IP antibody was anti-Ago2 (ab186733, Abcam) and anti-YTHDF2 (ab246514, Abcam).

### FISH assay

Cy3 labeled LINC01273 and FAM labeled miR-600 probes were designed and synthesized by GenePharma (Shanghai, China). Cell lysates were collected and incubated with the above probes, FISH assays were conducted using Fluorescent In Situ Hybridization Kit (Cat.F11201, GenePharma) as per the manufacturer’s protocol. The fluorescence signal was observed under a microscope.

### Luciferase reporter assay

The LINC01273 sequence with wild-type or mutant m^6^A site was ligated into the psiCHECK-2 dual-luciferase reporter vector (Promega, Madison, WI, USA). Then, METTL3-overexpressing pcDNA 3.0 plasmid was co-transfected with the above psiCHECK-2 vector into HCC cells. 48 h after transfection, the luciferase activity was tested by dual luciferase reporter system (Promega).

### Nude mice study

A total of 15 nude mice were obtained from the Vital River Laboratory (Beijing, China) and housed 5 mice per cage with free access to water and a normal chow diet. Huh7 cells with or without sorafenib resistance were subcutaneously injected into nude mice. The nude mice then grew naturally for 4 weeks, and the subcutaneous tumor volume was measured every week. After the experiment, tumor tissues were dissected, weighed, and photographed, followed by qRT-PCR and Western blot assays testing gene and protein expression, respectively.

### Statistical analysis

Tukey’s test or Student’s t-test for the unpaired results were used to evaluate the differences among more than three groups or between two groups, respectively. All data were generated using Graphpad Prism software. *P* < 0.05 was considered statistically significant.

## Results

Herein, we conducted a series of assays to clarify the role of LINC01273 in HCC. LINC01273 was identified as an oncogene and promoted sorafenib resistance via regulation of the newly identified LINC01273/miR-600/METTL3 feedback axis. Moreover, we found that LINC01273 could be used as a prognostic marker for HCC patients.

### LINC01273 is linked to sorafenib resistance in HCC

**Figure 1.**
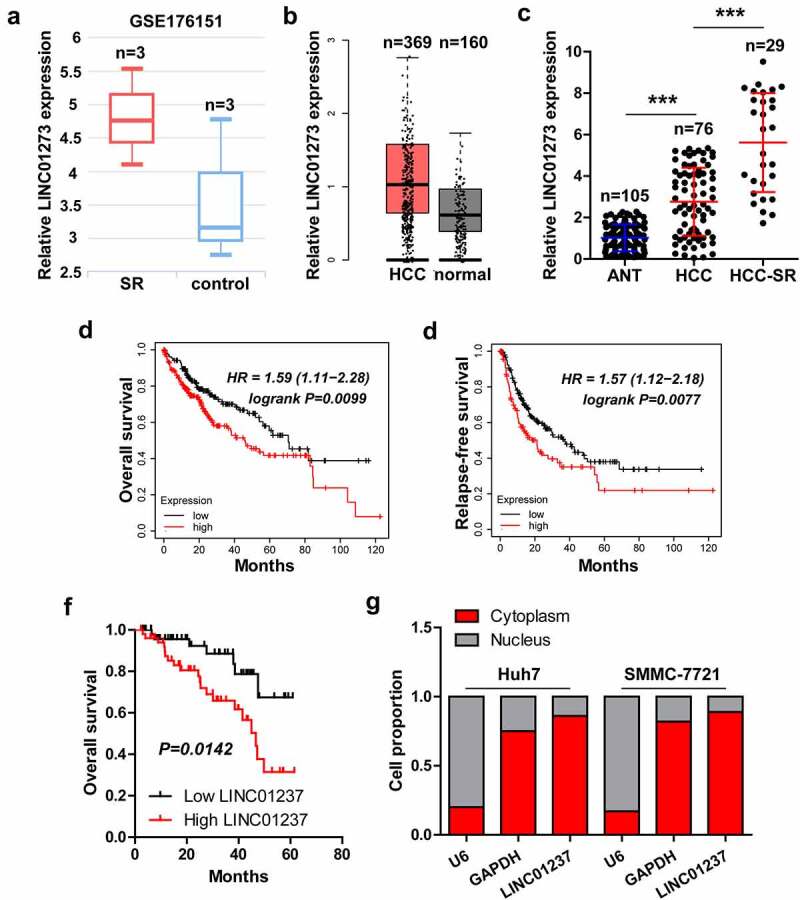
LINC01273 is a lncRNA related to sorafenib resistance. A-C. The expression of LINC01273 in GSE176151, GEPIA and our cohort. D-F. The survival curve of HCC patients based on LINC01273 level in KM plotter database (http://kmplot.com) and in our cohort. G. qRT-PCR analysis of LINC01273 level in Huh7 and SMMC-7721 cells, U6 and GAPDH were used as nuclear and cytoplasmic reference fragments, respectively. SR = sorafenib resistance; ANT = adjacent normal tissue; ***P < 0.001.

By analyzing the whole-transcriptome sequencing data between control and sorafenib-resistant HCC cells (GSE176151), we found that LINC01273 was the most differentially expressed lncRNA (Figure 1(a)). The GEPIA containing RNA sequencing expression data from TCGA and GTEx projects showed that LINC01273 had an upregulated trend in HCC as compared to normal tissues (Figure 1(b)). A total of 105 paired HCC tissues were collected (Figure S1), and found that LINC01273 was indeed highly expressed in HCC tissues, especially in sorafenib-resistant cases (Figure 1(c)). The survival data from Kaplan-Meier plotter tool showed that high LINC01273 was significantly associated with shorter overall and relapse-free survival time (Figure 1(d,e)). In our cohort, HCC patients with high LINC01273 displayed a poorer overall time than those with low LINC01273 (Figure 1(f)). By analyzing the lncATLAS database, we found that LINC01273 was mainly expressed in the cytoplasm of some cancer cells, including HCC cells (Figure S2). The qRT-PCR results showed that LINC01273 was also predominantly located in the cytoplasm of Huh7 and SMMC-7721 cells (Figure 1(g)).

### Knockdown of LINC01273 attenuates sorafenib resistance

**Figure 2.**
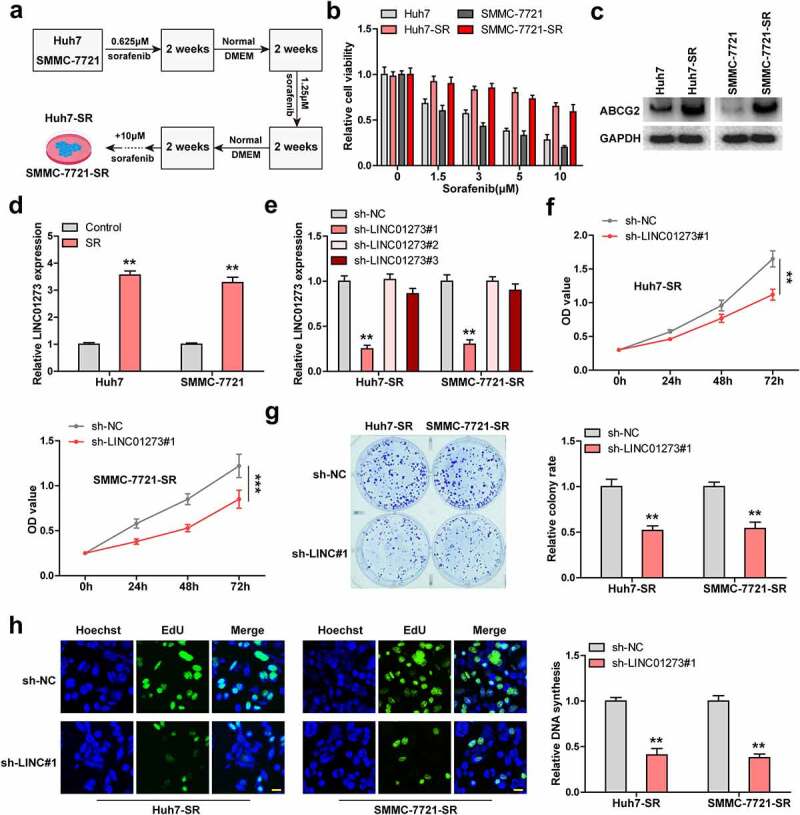
Knockdown of LINC01273 reduces sorafenib resistance. A. The flowchart of constructing sorafenib resistant cell lines. B. CCK-8 testing cell viability of the indicated cells after treatment with different concentrations of sorafenib. C. Western blot testing the level of ABCG2 expression in the indicated cells. D. qRT-PCR assay analyzing the expression of LINC01273 in sorafenib resistant cell lines. E. qRT-PCR verifying the silence efficiency of these shRNAs. F-H. CCK-8, colony formation and EdU assays detecting cell viability, colony and DNA synthesis rate in LINC01273-silenced sorafenib resistant cell lines. Scale bar = 50 μM, **P < 0.01.

We established sorafenib-resistant Huh7 and SMMC-7721 cells by long-term exposure to low to high concentrations of sorafenib, as shown in Figure 2(a). Cell viability of sorafenib-resistant cells was notably increased compared to control cells (Figure 2(b)). And ABCG2, a well-known marker of sorafenib-resistant cells [24], was significantly upregulated in sorafenib-resistant cells (Figure 2(c), Figure S3). As expected, Huh7 and SMMC-7721 cells with sorafenib resistance exerted higher LINC01273 levels compared to wild-type cells (Figure 2(d)). We designed four shRNAs targeting LINC01273, and found that sh-LINC01273#1 and sh-LINC01273#4 had the silencing effects (Figure S4a), which was then used for the subsequent assays (Figure 2(e)). The results of CCK-8 showed that knockdown of LINC01273 reduced cell viability of sorafenib-resistant HCC cells (Figure 2(f), Figure S4B, C). Likewise, less colony formation and EdU positive cells were observed in sorafenib-resistant cells with LINC01273 silencing than those in wild-type cells (Figure 2(g,h), Figure S4d).

### LINC01273 regulates sorafenib resistance via METTL3

**Figure 3.**
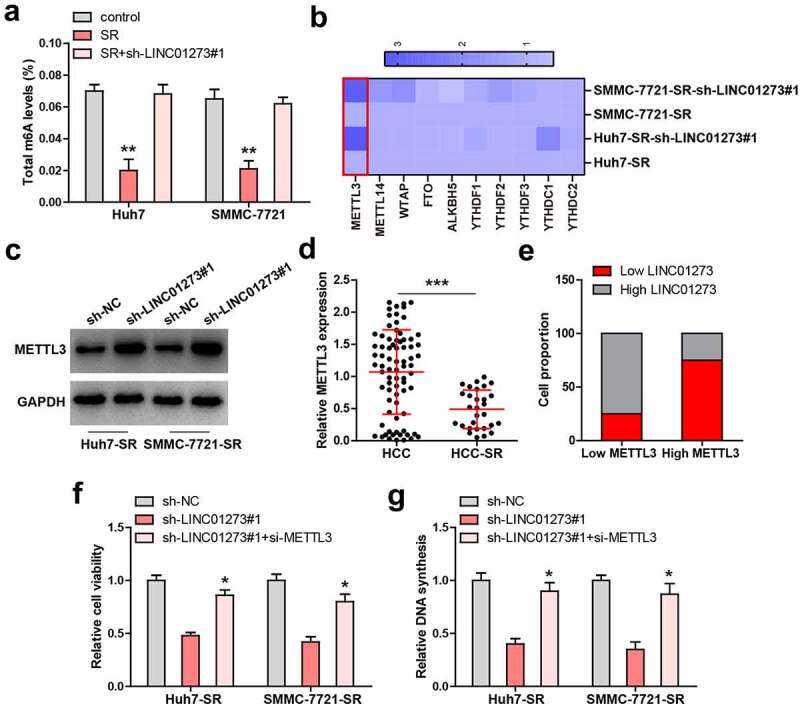
LINC01273 regulates METTL3 expression. A. Detection of total m^6^A levels in the indicated cell lines. B. qRT-PCR analysis of the m^6^A-related gene levels in LINC01273-silenced sorafenib resistant cell lines. C. Western blot testing METTL3 protein expression in LINC01273-silenced sorafenib resistant cell lines. D, E. qRT-PCR testing METTL3 expression in sorafenib resistant HCC tissues, followed by analysis of its correlation with LINC01273. F, G. Detection of cell viability and DNA synthesis rate in LINC01273-silenced sorafenib resistant cell lines with METTL3 silencing. *P < 0.05,**P < 0.01.

Given that m^6^A is involved in drug resistance, we then assessed total m^6^A level. The results showed that total m^6^A was significantly decreased in sorafenib-resistant HCC cells (Figure 3(a)); however, knockdown of LINC01273 almost completely rescued m^6^A level (Figure 3(a)). We then tested several m^6^A-related factors, and found that only METTL3 expression was significantly altered in both sorafenib-resistant Huh7 and SMMC-7721 cells with LINC01273 knockdown (Figure 3(b)). Similarly, the METTL3 protein level displayed the same trend (Figure 3(c), Figure S5). Moreover, METTL3 expression was significantly downregulated in sorafenib-resistant HCC tissues (Figure 3(d)), and 75% of HCC patients with high LINC01273 expression showed low METTL3 level (Figure 3(e)). Functionally, the reduced cell viability and DNA synthesis rate caused by LINC01273 silencing in sorafenib-resistant Huh7 and SMMC-7721 cells were remarkably rescued after knockdown of METTL3 (Figure 3(f, g)).

### LINC01273 reduced METTL3 level via miR-600

**Figure 4.**
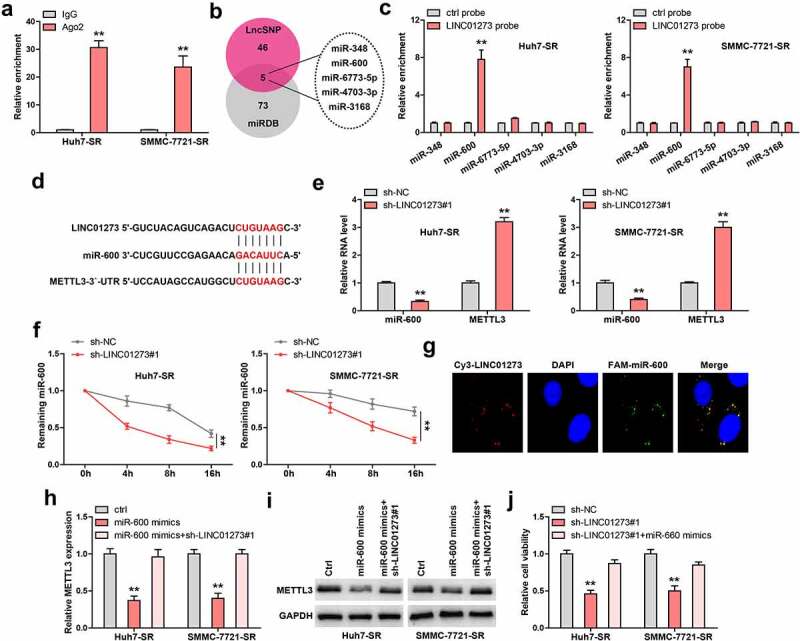
LINC01273 binds to miR-600. A. RIP assay testing the enrichment of LINC01273 by Ago2. B. The intersection results of LncSNP and miRDB. C. RNA pull-down assay testing miRNA enrichment by LINC01273. D. The binding sites between LINC01273/METTL3 and miR-600. E. qRT-PCR testing the effects of LINC01273 on METTL3 and miR-600 levels. F. qRT-PCR testing miR-600 level in LINC01273-silenced cells after treatment with Actinomycin D at the different time. G. FISH assay testing the co-location between LINC01273 and miR-600. H, I. METTL3 expression in miR-600-overexpressing cells with LINC01273 knockdown. J. CCK-8 testing cell viability in LINC01273-silenced cells transfected with miR-600 mimics. **P < 0.01.

In light of the cytoplasmic location of LINC01273, we conjectured that LINC01273 may regulate METTL3 via sponging miRNA. As expected, the Ago2-RIP results showed that LINC01273 was abundantly enriched by Ago2, a core component of RNA-induced silencing complex (RISC) (Figure 4(a)). Then, we searched for the common targeting miRNAs of LINC01273 and METTL3 using LncSNP and miRDB (Figure 4(b)). A total of 5 miRNAs were predicted (Figure 4(b)), but the results of RNA pull-down showed that only miR-600 was significantly enriched by LINC01273 in these two cells (Figure 4(c)). The binding sequences of LINC01273, miR-600, and METTL3 are shown in Figure 4(d). Further, LINC01273 knockdown resulted in a significant decrease in miR-600 expression, but was accompanied by an increase in METTL3 level (Figure 4(e)). Importantly, the half-life of miR-600 was shortened by nearly 4 h after silencing of LINC01273 (Figure 4(f)). Thus, we speculated that LINC01273 increases miR-600 stability via directly sponging effect. Consistently, FISH assay showed that LINC01273 was co-localized with miR-600 in the cytoplasm (Figure 4(g)). In addition, miR-600 overexpression reduced the luciferase activity of reporter containing METTL3 3`-UTR, whereas mutation of binding site abolished this effect (Figure S5). Moreover, the METTL3 mRNA and protein levels were significantly decreased in miR-600-overexpressing sorafenib-resistant Huh7 and SMMC-7721 cells, but these effects were entirely blocked by LINC01273 knockdown (Figure 4(h,i), Figure S7a). Functionally, the weakened cell viability induced by LINC01273 knockdown was partly rescued after miR-600 overexpression (Figure 4(j)).

### LINC01273 is regulated by m^6^A

**Figure 5.**
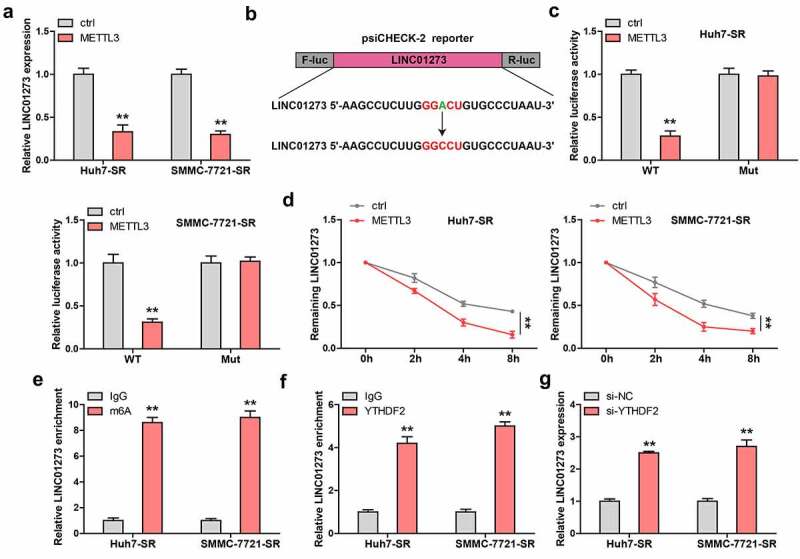
LINC01273 is modified by m^6^A induced by METTL3. A. The effect of METTL3 overexpression on LINC01273 expression. B, C. The m^6^A motif on LINC01273 was mutated and inserted into psiCHECK-2 vector, followed by luciferase reporter assay in cells transfected with METTL3 plasmid. D. qRT-PCR testing LINC01273 level in METTL3-overexpressed cells after treatment with Actinomycin D at the different time. E. MeRIP assay analyzing m^6^A enrichment on LINC01273. F. RIP assay testing the enrichment of LINC01273 by YTHDF2. G. qRT-PCR analysis of LINC01273 expression in sorafenib resistant cell lines transfected with YTHDF2 siRNA. **P < 0.01.

Given that m^6^A controls lncRNA expression, we then wondered whether LINC01273 was controlled by METTL3, thus forming a regulatory loop. Interestingly, LINC01273 was dramatically decreased in sorafenib-resistant HCC cells transfected with METTL3 expressing plasmid (Figure 5(a)). By analyzing the full-length of LINC01273, we found only one m^6^A site with very high confidence, the adenine was replaced by cytosine, and luciferase reporter assay was carried out (Figure 5(b)). The results show that METTL3 overexpression significantly reduces the luciferase activity of wild-type vector, but not that of mutant one (Figure 5(c)). Importantly, the half-life of LINC01273 was notably decreased by about 2 h after exogenous expression of METTL3 (Figure 5(d)). As shown in Figure 5(e,f), LINC01273 was enriched with m^6^A and YTHDF2, a m^6^A ‘reader’ affecting RNA stability. The silencing of YTHDF2 remarkably increased LINC01273 level in sorafenib-resistant HCC cells (Figure 5(g)).

### Knockdown of LINC01273 limits sorafenib resistance in vivo

**Figure 6.**
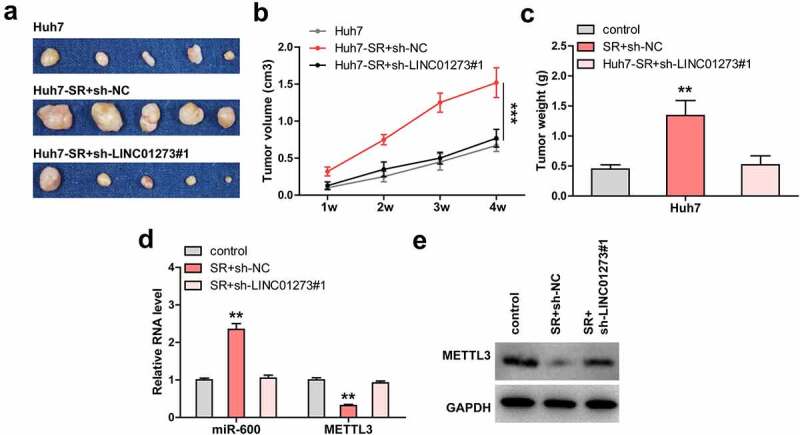
LINC01273 knockdown restores the sensitivity of HCC cells to sorafenib *in vivo*. A-C. Tumor image, volume and weight in the indicated groups. D, E. Analysis of miR-600 and METTL3 levels in the indicated groups using qRT-PCR and Western blot. **P < 0.01, ***P < 0.001.

Lastly, Huh7, Huh7-SR, and Huh7-SR+sh-LINC01273#1 cells were subcutaneously injected into nude mice. The results showed that tumors from sorafenib-resistant Huh7 cells were significantly larger than those from wild-type Huh7 cells (Figure 6(a-c)). However, when LINC01273 was knocked down, the tumor size almost returned to the control level (Figure 6(a-c)). As expected, miR-600 level was increased and METTL3 was decreased in sorafenib-resistant group as compared to control group (Figure 6(d,e), Figure S7b), but in Huh7-SR+sh-LINC01273#1 group, the expression of miR-600 and METTL3 was similar to that in control Huh7 group (Figure 6(d,e), Figure S7b).

## Discussion

In the present study, we characterized a lncRNA controlling the sensitivity of HCC cells to sorafenib. High LINC01273 was observed in HCC and sorafenib-resistant tissues, which was related to dismal prognosis. We established the sorafenib-resistant Huh7 and SMMC-7721 cells, and found that knockdown of LINC01273 notably restored the response of the HCC cells to sorafenib, and sorafenib-resistant cell proliferation and tumor growth were effectively controlled by depletion of LINC01273. In-depth mechanism research revealed that LINC01273 was co-located with miR-600 in the cytoplasm of HCC cells, LINC01273 increased miR-600 stability via acting as a ‘reservoir’, enhancing the repressive effect of miR-600 on METTL3 mRNA, resulting in METTL3 downregulation, thereby conferring the sorafenib resistance of HCC cells. In turn, LINC01273 was modified with m^6^A, and METTL3 increased m^6^A level of LINC01273, reducing the stability of LINC01273 following YTHDF2 recognition. Hence, the regulatory feedback between LINC01273 and METTL3 was critical and deregulated in sorafenib-resistant HCC cells. Taken together, we uncover a crosstalk of lncRNA, m^6^A modification, and sorafenib resistance, and provide insights into the multiple molecular mechanisms of sorafenib resistance in HCC, as well as extending the understanding of therapy resistance.

LncRNA has many biological functions, the most well known of which is its role as a molecular sponge for miRNA [25]. miRNA is a class of non-coding single-stranded RNA molecules with a length of about 22 nucleotides encoded by endogenous genes, which are involved in the regulation of post-transcriptional gene expression in animals and plants [26]. The function of miRNA depends on the RISC complex, which directs miRNA to target mRNA, promotes degradation of target genes, or inhibits translation [27]. Generally, lncRNA and miRNA sequences complement each other, sequestering the inhibitory effect of miRNA on target genes, thus indirectly increasing gene expression [28]. For instance, FAM225B directly bound to miR-613, a miRNA targeting CCND2, resulting in CCND2 upregulation, facilitating nasopharyngeal carcinoma tumorigenesis and metastasis [29]. PENG was expressed at a lower level in clear cell renal cell carcinoma, it inhibited tumor cell growth by increasing PDZK1 via sponging miR-15b [30]. However, some recent studies have shown that lncRNA do not always play the opposite role to miRNA, it can act as a protective agent for miRNA, thus making miRNA more stable in inhibiting the function of target genes, such as DNM3OS [31], SAF [32] and H19 [33]. To date, the mechanism of lncRNA protecting miRNA has not been clarified, which may be related to different tertiary structures, and which molecules participate in this process needs to be further explored.

Herein, we found that LINC01273 was located in the cytoplasm and prolonged the half-life of miR-600 by sequence complementation, resulting in miR-600 accumulation, accompanied by downregulation of METTL3 expression, a m^6^A ‘writer’ that enhances the sensitivity of HCC cells to sorafenib via regulation of autophagy [20]. Thus, our results suggest that LINC01273 is a ‘reservoir’ for miR-600 in HCC cells, and the pattern of lncRNA’s protective effect on miRNA may be far greater than what we currently understand, which requires further investigation. Furthermore, we have also verified the regulatory signaling of LINC01273/miR-600/METTL3 *in vivo*, suggesting that dysregulation of LINC01273/miR-600/METTL3 axis may be responsible for sorafenib resistance in HCC cells.

m^6^A is the most common and abundant post-transcriptional modifications, which exists not only on mRNA but also on tRNA and rRNA [34,35]. Recently, m^6^A has been proposed to be involved in RNA splicing, translation, stability, and epigenetic effects of some ncRNAs [36,37]. METTL3, as a m^6^A methyltransferase, participates in the regulation of lncRNA. For example, METTL3 increased m^6^A modification of PCAT6, resulting in PCAT6 upregulation in an IGF2BP2-dependent manner [38]; LCAT3 stability was significantly increased in a METTL3-dependent manner in lung adenocarcinomas, thereby activating MYC transcription via FUBP1 [39]. Here, we found that LINC01273 was also modified with m^6^A and controlled by METTL3, METTL3 reduced LINC01273 stability via increasing m^6^A level of LINC01273, followed by recognition by YTHDF2, a m^6^A ‘reader’ destabilizing m^6^A-containing RNA through direct recruitment of the CCR4-NOT deadenylase complex [40–42]. Thus, a previously unappreciated METTL3/YTHDF2/m^6^A-LINC01273 was identified in HCC cells with sorafenib resistance, and the regulatory loop between METTL3 and LINC01273 amplified the effect of LINC01273 in promoting sorafenib resistance. Of note, METTL3 is frequently reported to play an oncogenic role in HCC [43]; however, in agreement with a recent study showing that METTL3 depletion contributes to the sorafenib resistance in HCC [20], our data also identified METTL3 as a negative regulator of sorafenib resistance. This may be explained by the bidirectional effect of METTL3, which may depend on the context, and the underlying mechanism is worthy of in-depth study.

There are some limitations in our study, among which the most important one is that the enrolled samples in this study are all retrospective and there may be some potential bias. Prospective research methods should be adopted in the subsequent study.

Emerging evidence suggests that lncRNA is a promising HCC therapeutic target [44], lncRNA targeting method has some advantages over protein targeting method because the base pairing principle is more direct than designing a specific protein-binding inhibitor. Antisense oligonucleotides (ASOs) and RNA interference (RNAi) treatment has already been applied in the treatment of hepatitis B virus (HBV) [45]. Therefore, targeting LINC01273 for the treatment of sorafenib-resistant HCC is feasible and promising.

## Conclusions

Collectively, our findings for the first time demonstrate that LINC01273 is a novel driver of sorafenib resistance in HCC cells. Targeting of LINC01273 may be an optional promising treatment for sorafenib-resistant HCC patients.

## Supplementary Material

Supplemental Material

## Highlights

- LINC01273 is upregulated in HCC and is associated with sorafenib resistance.
- Knockdown of LINC01273 reduces sorafenib resistance.
- LINC01273 regulates METTL3 level via miR-600.
- LINC01273 is modified by m^6^A induced by METTL3.
- LINC01273 knockdown restores the sensitivity of HCC cells to sorafenib *in vivo*.
