# Supplementary material for: Long intergenic non-protein coding RNA 1273 confers sorafenib resistance in hepatocellular carcinoma via regulation of methyltransferase 3
Source: Bioengineered. 2022 Jan 17;13(2):3108–21. doi: 10.1080/21655979.2022.2025701 (PMC8973700; doi:10.1080/21655979.2022.2025701)
Supplement: Supplemental Material [file KBIE_A_2025701_SM4793.zip › supplementary/Supplementary figures.doc]

**Figure S1.** The morphology of representative normal liver and HCC in our study.

**Figure S2.** LncATLAS showing the location of LINC01273 in different cells.

**Figure S3**. Quantification of ABCG2 protein in Huh7 and SMMC-7721 cells with sorafenib resistance. ***P*<0.01.

**Figure S4**. The shRNA#4 targeting LINC01273 was designed, and CCK-8 and colony formation assays testing the effect of LINC01273 knockdown on cell viability and colony formation ability, respectively. ***P*<0.01, ****P*<0.001.

**Figure S5**. Quantification of METTL3 protein in Huh7 and SMMC-7721 cells with sorafenib resistance. ***P*<0.01.

**Figure S6**. The effects of miR-600 overexpression on the luciferase activity of wild-type and mutant METTL3 3`-UTR reporters. ***P*<0.01.

**Figure S7**. Quantification of METTL3 protein in sorafenib-resistant Huh7 and SMMC-7721 cells transfected with miR-600 mimics (A) and in subcutaneous tumor tissues (B). ***P*<0.01.
